# RHO to the DOCK for GDP disembarking: Structural insights into the DOCK GTPase nucleotide exchange factors

**DOI:** 10.1016/j.jbc.2021.100521

**Published:** 2021-03-05

**Authors:** Andrew P. Thompson, Christina Bitsina, Janine L. Gray, Frank von Delft, Paul E. Brennan

**Affiliations:** 1 Nuffield Department of Medicine, Alzheimer’s Research UK Oxford Drug Discovery Institute, University of Oxford, Oxford, United Kingdom; 2 Nuffield Department of Medicine, Target Discovery Institute, University of Oxford, Oxford, United Kingdom; 3 Nuffield Department of Medicine, Centre for Medicines Discovery, University of Oxford, Oxford, United Kingdom; 4 Diamond Light Source, Harwell Science and Innovation Campus, Didcot, United Kingdom; 5 Department of Biochemistry, University of Johannesburg, Auckland Park, South Africa

**Keywords:** dedicator of cytokinesis (DOCK), guanine nucleotide exchange factor, guanosine triphosphate (GTP), Ras homologous (RHO) small GTPases, cell signaling, structural biology, drug discovery, AD, Alzheimer’s disease, aPKC, atypical protein kinase, APP, amyloid precursor protein, CDOCK, dedicator of cytokinesis, GDI, GDP dissociation inhibitor, GDP, guanosine diphosphate, GEF, guanine nucleotide exchange factor, GTP, guanosine triphosphate, MRCK, myotonic dystrophy kinase-related CDC42-binding kinase, PAK, p21-activated kinase

## Abstract

The human dedicator of cytokinesis (DOCK) family consists of 11 structurally conserved proteins that serve as atypical RHO guanine nucleotide exchange factors (RHO GEFs). These regulatory proteins act as mediators in numerous cellular cascades that promote cytoskeletal remodeling, playing roles in various crucial processes such as differentiation, migration, polarization, and axon growth in neurons. At the molecular level, DOCK DHR2 domains facilitate nucleotide dissociation from small GTPases, a process that is otherwise too slow for rapid spatiotemporal control of cellular signaling. Here, we provide an overview of the biological and structural characteristics for the various DOCK proteins and describe how they differ from other RHO GEFs and between DOCK subfamilies. The expression of the family varies depending on cell or tissue type, and they are consequently implicated in a broad range of disease phenotypes, particularly in the brain. A growing body of available structural information reveals the mechanism by which the catalytic DHR2 domain elicits nucleotide dissociation and also indicates strategies for the discovery and design of high-affinity small-molecule inhibitors. Such compounds could serve as chemical probes to interrogate the cellular function and provide starting points for drug discovery of this important class of enzymes.

## GTPase function and regulation

**Figure 1:**
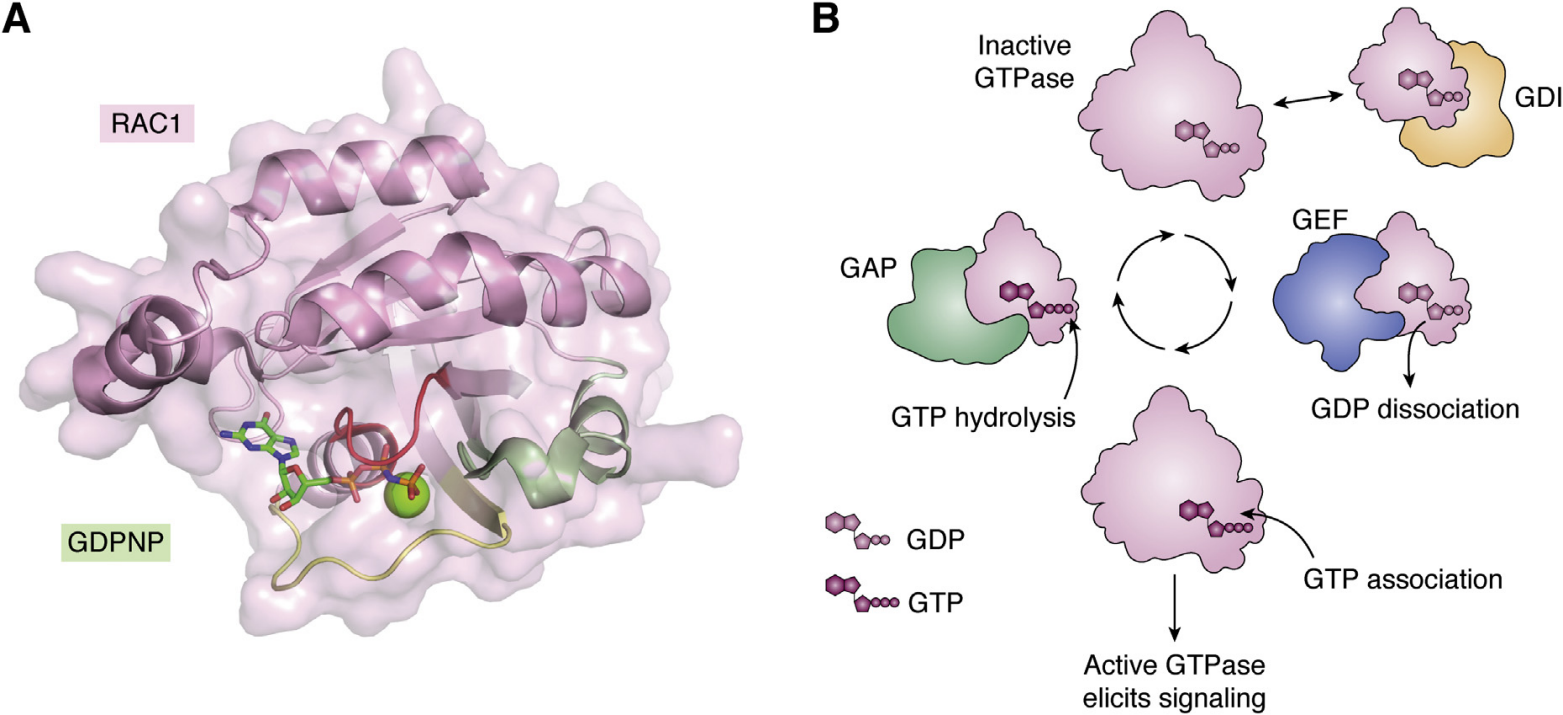
***A*, RHO family small GTPase RAC1 (*pink surface*, *cartoon*; PDB ID: 3TH5) bound to phosphoaminophosphonic acid–guanylate ester (GDPNP; *green sticks*) and magnesium (*green sphere*).** The phosphate-binding loop (P-loop; *red ribbon*; residues 10–17) binds the phosphates of guanosine polyphosphates and magnesium. Switch loops 1 and 2 (*yellow* and *green ribbons*; residues 27–40, 57–74 respectively) change conformation depending on the presence of GDP or GTP to affect cellular signaling. *B*, GTPases (*pink*) cycle between an inactive, GDP-bound state to an active, GTP-bound state. The switch loop conformations in the active state allow GTPases to bind to and elicit cellular signaling processes. GDP dissociation is prevented by guanosine dissociation inhibitors (GDIs, *orange*) and accelerated by guanosine nucleotide exchange factors (GEFs, *blue*), while GTP hydrolysis is induced by GTPase-activating proteins (GAPs). Together these interactions tightly control the location and timing of GTPase activity. Figure created with BioRender.com.

The RAS superfamily of over 150 small GTPases is comprised of ∼20 kDa monomeric G-proteins, which are divided into five main families: RAS, RHO, RAN, RAB, and ARF based on structural and functional conservation (1, 2). The five families share the same basic biochemical function as binary molecular switches that affect a wide array of signaling cascades, eliciting cellular functions such as gene expression, protein and vesicle transport, and cytoskeletal remodeling (3, 4, 5). The latter of these is the most well-known function of the RAS-homologous (RHO) GTPase family. Through organization of actin structures in the cell, RHO GTPase-controlled signaling pathways dictate cellular motility and proliferation. Such signaling cascades are modulated through RHO GTPase effector families, examples of which include the atypical protein kinase Cs (aPKCs), myotonic dystrophy kinase-related CDC42-binding kinases (MRCKs), p21-activated kinases (PAKs), and RHO-associated protein kinases (ROCKs) (5). GTPases are referred to as binary molecular switches as they cycle between an active, guanosine triphosphate (GTP)-bound state and an inactive, guanosine diphosphate (GDP)-bound state (6). In the active state, GTPase signaling is conferred by conformational changes within the switch 1 and 2 loops (Fig. 1*A*), which dictate binding to effector proteins (7). Transition between these states is caused by hydrolysis of GTP to form GDP and dissociation of GDP to allow a new GTP molecule to bind. The affinity of the interaction between GTPases and either guanine nucleotides is extremely high (*K*_d_ 10^−7^–10^−11^) ensuring that these are committed states that do not spontaneously interchange; they do not respond to changes in cellular nucleotide concentrations (8). Rather, due to the intrinsically low GTPase activity of G-proteins, regulatory proteins are required for spatiotemporal control of the transition between active and inactive states (Fig. 1*B*). These regulatory proteins are therefore critical regulators of subsequent signaling cascades, ensuring that they occur at certain cellular and subcellular locations and at appropriate times in response to environmental stimuli. There are three categories of GTPase regulating molecules: guanine nucleotide exchange factors (GEFs), which are responsible for facilitating GDP dissociation, allowing subsequent GTP loading; GTPase activating proteins (GAPs) that stimulate GTP hydrolysis to result in an inactive, GDP bound GTPase; and GDP dissociation inhibitors (GDIs), which act only on the RHO and RAB subfamilies to maintain an inactive GTPase state in the cytoplasm (6). The localized modulation of GTPases by these mediators varies between cell types and thus constitutes a complex network of regulatory mechanisms (9). In particular, GEF-catalyzed nucleotide exchange results in an active GTPase that can transduce signals to control cellular functions (10). As evidenced by reports of small molecules targeting the RAS GEF SOS1, inhibition of GEF activity would thus arrest the given GTPase signaling pathways, constituting a promising target for small-molecule therapeutic development (11, 12).

## Disease associations of the RHO GTPase family

Due to their pleiotropic nature, aberrant signaling activity of GTPases has been associated with many disease phenotypes. This is best demonstrated by the observation that RAS proto-oncogenes HRAS, NRAS, and KRAS are mutated in almost 30% of human cancer types (13, 14, 15, 16). However, overactivation of the RHO subfamily such as RAS-related C3 botulinum toxin substrate 1 (RAC1) and cell division cycle 42 homolog (CDC42) are also known to promote the hyperactivation of critical signaling cascades leading to cancer (10, 13, 17). In mammals, the RHO family consists of 20 members in total, further distributed into eight subfamilies based on sequence homology: CDC42, RAC, RHO, RHOBTB, RHOD/F, RHOH, RHOU/V, and RND (9). Among the RHO molecular switches, RAC1, CDC42, and RHOA are the most well defined and characterized (18, 19). Further investigation into the remaining RHO GTPases is required to determine the homeostatic and disease associations of this crucial protein family.

RHO GTPases also modulate neuronal functions, as evidenced by the implication of their dysregulation in neurological disorders. By coordinating actin cytoskeletal rearrangements (20, 21, 22), RHO GTPases elicit cellular adhesion and migration activities in neural tissues, regulating neuronal morphogenesis (18, 23, 24) and dendrite elaboration (25, 26) by participating in several signal transduction pathways through downstream effectors such as neuronal Wiskott–Aldrich syndrome protein (N-WASP) (26, 27). Improper regulation in these signaling cascades disrupts homeostasis, possibly leading to neurodegeneration (16, 28). This can be inferred from mouse primary hippocampal neuronal experiments, where RAC1 inhibition with compound NSC23766 leads to attenuated γ-secretase activity, resulting in a reduction in cellular amyloid precursor protein (APP) and subsequent β-amyloid plaque formation, a hallmark of Alzheimer’s disease (AD) (29). Mounting evidence also connects RHOA, RAC1, and CDC42 to AD phenotypes such as synaptic dysfunction, dendritic spine loss, cytoskeletal abnormalities, and cell cycle re-entry (16). Similarly, emerging evidence supports that deregulation of RHO GTPase signaling has a significant impact toward the development of autism spectrum disorders (ASDs) (23). Considering the growing body of RHO GTPase disease associations, further exploration into the cellular and molecular modulation of RHO GTPases is warranted.

## DH and DOCK RHOGEFs

RHO GTPases can be activated by two distinct classes of GEFs: the Dbl homology (DH) domain containing enzymes and the dedicators of cytokinesis (DOCK). DH GEFs constitute the larger of the two classes, with over 70 members (30, 31). The eponymous domain is often associated with a pleckstrin homology (PH) domain, forming the DH/PH domain architecture that confers GEF activity. In contrast, there are only 11 DOCK proteins, and they are characterized by two structurally conserved domains: DOCK homology regions 1 and 2 (DHR1, DHR2) instead of the canonical DH/PH domain structure of the larger RHO GEF family.

**Figure 2:**
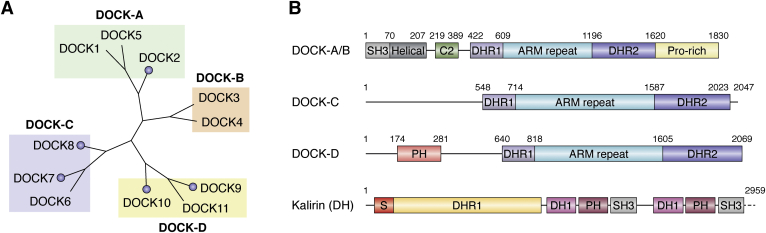
***A*, DOCK proteins are largely classified into their subfamilies based on phylogeny, as well as sequence and substrate specificity.** DOCK proteins with publicly available structural information are indicated by a *circle*. *B*, domain architecture of the DOCK proteins also follows their subfamilial categorization. All DOCK proteins contain a DHR1 as well as the catalytic DHR2 domain. Domains and numbering for the DOCK-A and B are based on the recently published Cryo-EM structure of the full-length DOCK2. The ARM repeat is putatively present in all DOCK proteins based on structural homology prediction. Domain numbering is represented by DOCK6 and 9 for DOCK-C and D subfamilies respectively. The domain architecture for Kalirin is provided as an example Dbl-Homology GEF. Kalirin is truncated for clarity of comparison (represented by *dashed line*). The canonical DH/PH architecture present twice in Kalirin confers its GEF activities.

**Figure 3:**
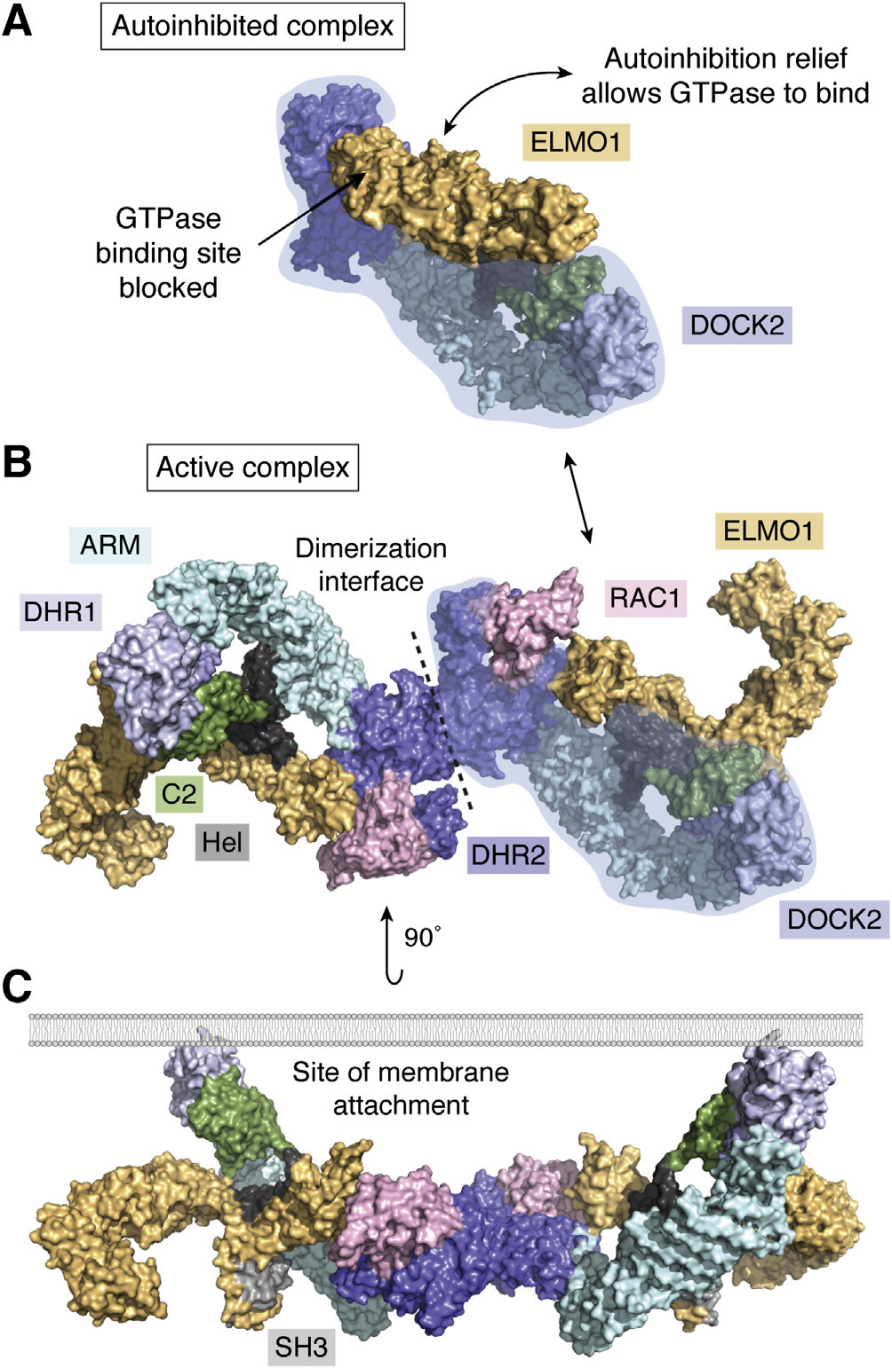
***A*, cryo-EM structure of the autoinhibited DOCK2-ELMO1 complex (PDB ID: 6TGC).** The DOCK2 (monomer depicted by transparent *blue highlight*) GTPase-binding site is occupied by ELMO1 (*yellow*). *B*, cryo-EM structure of the active DOCK2-ELMO-RAC1 complex (PDB ID: 6TGC). DOCK2 dimerizes through the DHR2 domain (*blue*), which also binds RAC1 (*pink*). ELMO1 binds to the SH3, Helical (Hel, *black*), C2 (*green*), and DHR2 domains, as well as the C-terminal pro-rich sequence (not depicted). ELMO1 undergoes conformational change upon relief of the autoinhibitory state depicted in panel *A*. *C*, the entire complex is localized to the membrane through the phospholipid-binding capabilities of the DOCK2 DHR1 and C2 domains. Complex is rotated 90 degrees out of the page with respect to *B*.

The DHR1 domain is a C2-like domain that binds phospholipids to target DOCK complexes to the membrane, such as at a leading cellular edge where signaling can be initiated to drive motility (32). The DHR2 domain is responsible for GEF activity in a mechanism that is distinct from that of DH-containing GEFs (33, 34, 35). Based on sequence homology, phylogeny, and substrate specificity, the DOCK family is classified into four subfamilies: DOCK-A (DOCK1, 2, 5), DOCK-B (DOCK3, 4), DOCK-C (DOCK6, 7, 8), and DOCK-D (DOCK9, 10, 11) (Fig. 2). Generally DOCK families A and B contain N-terminal SRC Homology 3 (SH3) domains and a C-terminal proline-rich region that bind each other to maintain the protein in an autoinhibited state, an interaction that is alleviated upon binding adaptor proteins such as engulfment and cell motility protein 1 (ELMO1) (36, 37). Crystallographic and NMR studies on recombinant domains of these proteins culminated in a hypothetical model of the overall DOCK2-RAC1-ELMO1 signaling complex. A recent cryo-electron microscopy-derived structure of this signaling complex using the full-length proteins is consistent with these predictions and provides the first high-resolution view of the conformational and phosphorylation events leading to activation of the complete complex (Fig. 3) (38). The region interjoining the DHR1 and 2 domains has been identified as a series of armadillo (ARM) and Huntington, Elongation Factor 3, PR65/A, TOR (HEAT) repeats in DOCK2, termed the DOCK2-ARM region. Structural prediction indicates that this region is structurally conserved throughout the DOCK family, based on similarity to the aforementioned cryo-EM structure of DOCK2, RAC1, and ELMO1 (summarized in Fig. 2*B*; structural prediction performed in HHpred server) (39). An additional C2 domain N-terminally adjacent and in addition to the DHR1 was also observed in DOCK2 in this structure. Structural prediction indicates that this observed region N terminal to the DHR1 domain is conserved in the DOCK-A and B subfamilies, but differs within the C and D proteins (Fig. 2*B*) (39). Unlike the DOCK-A and B proteins, DOCK-C and D members do not contain the SH3 domain and therefore do not bind the ELMO1 adapter. There are no reports of DOCK-C and D family members interacting with such adapters to alleviate autoinhibition; however, multiple interacting partners have been observed for other members of the DOCK family, activating them in other ways such as *via* dephosphorylation (40). Uniquely, the DOCK-D proteins contain a PH domain, which, similarly to the DHR1 domain, is involved in membrane localization through phospholipid binding (34).

GTPase substrate specificities are broadly distinct in DOCK subfamilies. Substrate binding is conferred by specific residues within the DHR2 domain (41, 42). Generally, DOCK-A and DOCK-B proteins activate the RAC GTPases specifically (35, 41), whereas the DOCK-D subfamily is responsible for activating CDC42. The DOCK-C subfamily has dual binding capability, activating both CDC42 and RAC1, except for DOCK8, which appears to be a specific CDC42 GEF (38). DOCK10 also appears to bind more than one GTPase as evidenced by recent crystal structures in complex with CDC42 and RAC3 (Table 1) (42, 43, 44). However, as research has mainly focused on the RHO GTPases RAC1 and CDC42, there may indeed be uncharacterized alternate substrates for the DOCK GEFs among the full complement of 20 RHO GTPases. As regulators of the pleiotropic RHO GTPases, DOCK GEFs exhibit varying biological function associations, cell-type expression profiles, and subcellular localization as discussed below.

**Table 1:** Available structural information for DOCK proteins

Dock protein	Dock domain	Partner protein	Ligand	PDB ID	Reference
DOCK1	DHR1			3L4C	Premkumar, L. *et al.* (32)
	SH3			2M0Y (NMR)	Liu, X. *et al.* (138)
DOCK2	DHR2	RAC1		2YIN	Kulkarni, K. *et al.* (41)
	DHR2	RAC1 (T17N mutant)		3B13	Hanawa-Suetsugu, K. *et al.* (37)
	SH3 peptide	ELMO1 peptide		2RQR (NMR)	Hanawa-Suetsugu, K. *et al.* (37)
	SH3	ELMO1		3A98	Hanawa-Suetsugu, K. *et al.* (37)
	Full length	ELMO1		6TGB (Cryo-EM)	Chang, L. *et al.* (38)
	Full length	RAC1, ELMO1		6TGC (Cryo-EM)	Chang, L. *et al.* (38)
DOCK7	DHR2	CDC42		6AJ4	Kukimoto-Niino, M. *et al.* (42)
	DHR2 (I1836Y Mutant)	CDC42		6AJL	Kukimoto-Niino, M. *et al.* (42)
DOCK8 (*MUS MUSCULUS*)	DHR2	CDC42 (T17N Mutant)		3VHL	Harada, Y. *et al.* (113)
DOCK9	PH			1WG7 (NMR)	Suetake, T. to be published
	DHR2	CDC42		2WM9	Yang, J. *et al.* (35)
	DHR2	CDC42	GDP	2WMN	Yang, J. *et al.* (35)
	DHR2	CDC42	GTP	2WMO	Yang, J. *et al.* (35)
DOCK10	DHR2	RAC3		6TM1	To be published
	DHR2	CDC42		6TKY	To be published
	DHR2	CDC42	GDP	6TKZ	To be published

## Biological function and disease associations of the DOCK GEFs

DOCK proteins control the spatiotemporal nature by which RHO GTPase signaling pathways occur. Therefore, the expression profiles and subcellular localization data reported in the literature and available on the Human Protein Atlas help explain the specific roles that each DOCK protein carries out (45, 46, 47). DOCK family functions in specific areas such as neurology (36, 48), immunology (49, 50), and cancer (17) have been extensively reviewed, and what follows is a summary of DOCK function with a focus on disease association of each family member (Table 2).

**Table 2:** Human DOCK RHO GEF protein family members

Protein	Tissue expression^a^	Subcellular localization^a^^,^^b^	Cellular function	Disease associations	Uniprot ID
DOCK1	Ubiquitous, elevated in brain, endocrine	N	Phagocytosis, migration	Metastasis in cancer	Q14185
DOCK2	Blood, bone marrow and lymphoid	C	Immune survival and migration	Immunodeficiency, Alzheimer’s disease	Q92608
DOCK3	Predominantly brain	C	Neuronal outgrowth	Developmental and motor disorders, Alzheimer’s disease	Q8IZD9
DOCK4	Brain, lung	N, C, P	Axon-dendrite polarity, migration	Autism, dyslexia, schizophrenia, metastasis in cancer	Q8N1I0
DOCK5	Predominantly in brain, lung and bone	C	Microtubule dynamics	Osteolysis	Q9H7D0
DOCK6	Ubiquitous	C	Actin remodeling, axon growth in CNS	Adams-Oliver syndrome, metastasis	Q96HP0
DOCK7	Brain, endocrine tissue, muscle, kidney	C	Axon formation, myelination	Glioblastoma, epileptic encephalopathy	Q96N67
DOCK8	Bone marrow, lymphoid tissue and blood	N, C	Immune survival and migration	Immunodeficiency, multiple sclerosis	Q8NF50
DOCK9	Ubiquitous	-	Neuronal synaptic plasticity	Papillary thyroid carcinoma	Q9BZ29
DOCK10	Brain, bone marrow, lymphoid tissue and blood	N, C	Innate cell growth and differentiation	Leukemia, multiple sclerosis, aging	Q96BY6
DOCK11	Bone marrow, lymphoid, adipose, female reproductive tissue	N	Filopodia formation, B cell signaling	Aging	Q5JSL3

a Data from Human Protein Atlas (https://www.proteinatlas.org).

b Nuclear (N) or Cytoplasmic (C) or Plasma membrane (P).

### DOCK1 (DOCK180)

DOCK1 is a GEF specific to the RAC subfamily of RHO GTPases. It is ubiquitously expressed, with elevated levels noted in the brain, endocrine, and epidermal tissues based on RNA expression provided in the Human Protein Atlas database, while protein expression data indicates higher levels in endocrine, female reproductive tissues, and lymphoid tissue. It has reported roles in cell migration, invasion, and phagocytosis (48, 51, 52, 53, 54). As has been demonstrated for several DOCK-A and B family proteins, DOCK1 autoinhibition is alleviated upon interaction with the ELMO adaptor proteins *via* the N-terminal SH3 domain, creating a complex that activates the RACs (36). DOCK1-mediated activation of RAC1 induces membrane ruffling, a formation of the motile cell surface consisting of actin filaments (55, 56). It has been reported that DOCK1 activity is crucial for migration in certain breast cancer cell lines, indicating that it is a potential target for general prevention of metastasis (51, 57). However, DOCK1 mutant zebrafish Schwann cells experienced developmental defects and myelination activities, indicating that DOCK1 is an important nervous system factor (58). While the ubiquitous expression of DOCK1, as well as its apparent importance to neural cell development, could pose problems when targeting tissue-specific disorders, inhibition of this GEF could have beneficial effects in cancer treatment.

### DOCK2 (KIAA0209)

DOCK2, responsible for activating RAC GTPases, is predominantly expressed in hematopoietic cells (59, 60, 61, 62). It is a crucial regulator of the immune system, controlling the activation of both adaptive and innate immune cells (50, 61, 63). This is illustrated in animal models, wherein genetic ablation of DOCK2 decreased the migration speed of T-cells and B-cells in lymphoid tissue (61). Multiple immunodeficiencies have also been linked to DOCK2 mutations (64). In the brain DOCK2 is exclusively expressed in microglia and has been identified as a biomarker of this cell type, as it is a molecular hub influencing both homeostatic and neural disease processes (65). It has been implicated in the pathogenesis of AD by enhancing amyloid beta (Aβ) plaque formation (66, 67). DOCK2 is consequently an interesting target for potential Alzheimer’s therapeutics. At the molecular level, DOCK2 translocation to the inner membrane surface leads to GTPase activation to signal for subsequent migration and differentiation. As previously mentioned, complexes of ELMO1 and DOCK2 with and without RAC1 were solved, providing further insight into this mechanism (Fig. 3) (38). Interactions of ELMO1 with RHOG and brain-specific angiogenesis G-protein-coupled receptors (BAI GPCRs), as well as phosphorylation of ELMO1 and DOCK2, lead to GEF activation. This insight may also be applicable to structurally conserved members of DOCK-A and B subfamilies. As a regulator of cell motility that is predominantly found in immune cells, DOCK2 is a promising target for anti-inflammation therapeutics. As highlighted above, an application of this could be in the treatment of AD, where there is a growing body of evidence implicating microglia in the progression of AD (68).

### DOCK3 (KIAA0299, modifier of cell adhesion, MOCA; presenilin-binding protein, PBP)

DOCK3, a GEF for RAC1, acts as regulator of actin reorganization in neuronal tissue (69, 70, 71, 72). DOCK3 participates in axonal and neurite outgrowth processes, particularly during early developmental stages *via* distinct signaling cascades that involve either the brain-derived neurotrophic factor-tyrosine kinase B (BDNF-TRKB)-mediated pathway or glycogen synthase kinase-3β (GSK-3β) (36, 69, 73, 74). DOCK3 gene variants leading to potentially nonfunctional protein were identified to cause developmental and motor issues in patients confirmed by a RAC1 pull-down assay and protein modeling (70). As indicated by its initial name, PBP, DOCK3 interacts with presenilin 1 (PS1), a catalytic unit of the γ-secretase proteolytic complex, which is responsible for processing amyloid precursor protein (APP) and subsequent formation and accumulation of β plaques (75, 76, 77, 78, 79). *In vitro* experiments have shown that induced expression of DOCK3 results in attenuated Aβ secretion through regulation of APP degradation (80, 81). The implications of the interaction between DOCK3 and PS1 in terms of AD progression are not fully understood and require further investigation. However, these findings highlight the role of DOCK3 in APP processing and axonal growth and the potential for modulating DOCK3-dependent signaling in the treatment for neurodegeneration.

### DOCK4 (KIAA07016)

DOCK4 activates RAC and RAP1 GTPases (82). The Human Protein Atlas RNA database indicates predominant expression in the brain and lung tissue, with low-to-moderate levels elsewhere. Signaling through DOCK4 and its partner GTPases controls cytoskeletal and morphological processes such as neuronal branching, polarization, and outgrowth as well as motility and invasion. Knockdown of DOCK4 with shRNA eliminates dendritic growth and branching in hippocampal neurons (83). DOCK4 variants leading to reduced GEF activity and subsequent deficits in neuronal morphology have been linked to neuropsychiatric disorders such as autism, dyslexia, and schizophrenia (84, 85). Indeed, DOCK4 polymorphism has been identified as a risk factor for these disorders in genetic studies (86, 87). The SH3 domain is crucial for the aforementioned neural processes. While the C-terminal proline-rich region is not essential for its regulating its catalytic activity, it has been reported to play a role in synaptic localization and mediating the interaction with the actin-binding protein cortactin (36, 84). Upregulation of DOCK4 expression was recently identified in placenta accreta spectrum, wherein cells are detrimentally hyperinvasive (88). DOCK4 is posited as a potential biomarker for invasion characteristics in breast cancer (89). Finally, DOCK4 was recently shown to contribute to low-density lipoprotein transport, a causative process in atherosclerosis progression (90).While these disease indications are promising in the treatment of invasive cancers or cardiovascular disease, the crucial role that DOCK4 plays in neural development implies that there is a delicate balance for homeostatic DOCK4 activity.

### DOCK5

DOCK5, one of the least studied family members, preferentially activates RAC1 over other RHO or RAC GTPases (91). It is predominantly present in the brain, lung, and bone tissues such as osteoclasts, but is also present in other tissues (92). One such tissue is the liver, where DOCK5 activity increases energy expenditure and insulin signaling by impeding mammalian target of rapamycin complex (mTORC1), linking DOCK5 downregulation to obesity (93). DOCK5-deficient mice, while otherwise healthy, were characterized by significantly increased bone mass, a classical symptom of improper bone resorption (94). This highlights DOCK5 as a potential target for inhibition to treat osteolytic diseases. In neurology, genome-wide association studies (GWAS) have identified this gene as a high-risk genetic factor associated with familial Parkinson's disease (PD) pathology (95). Finally, a recent study identified a link between a family with bipolar disorder and a novel DOCK5 mutation (96). The studies highlighted here constitute the basis for investigating the role of DOCK5 in multiple processes, including insulin signaling, bone homeostasis, and psychiatric disorders. Notably, DOCK5 activity in bone homeostasis appears to be unique to this family member; however, further research is required to determine the potential for therapeutically targeting DOCK5.

### DOCK6 (KIAA1395, ZIR1)

DOCK6 is reported to interact with both CDC42 and RAC1 (but not RHOA) in order to regulate actin remodeling, manifesting as neuron growth and regeneration in the brain (40, 43). Protein and RNA have been identified in the brain, endocrine, lung, gastrointestinal tract, liver and gall bladder, pancreas, kidney, reproductive tissues, adipose, and skin. Interestingly, DOCK6 preferentially activates RAC1 in dorsal root ganglion (DRG) neurons (36) despite the fact that it has been predicted to bind to CDC42 with higher affinity, suggesting that further investigation of this dual binding mode is required (97). Neuronal axon branching and extension are dependent on the phosphorylation state of DOCK6. Dephosphorylation by protein phosphatase 2A (PP2A) at Ser1194 activates GEF activity, resulting in axonal growth, whereas phosphorylation by AKT inactivates DOCK6 and causes branching to occur preferentially, as well as preventing axon regeneration (40). Four independent studies have identified mutations in the DOCK6 gene responsible for or associated with the development of the rare Adams–Oliver Syndrome (98, 99, 100, 101). Some of the observed mutations lead to the production of truncated DOCK6 proteins lacking the catalytic domains, while other mutations lead to point mutations in conserved residues (for example: V263D, E1052K). DOCK6 mutations are also associated with microcephaly, indicating that the protein may have a functional role during early development stages in the brain (69). As is commonly observed with DOCK proteins, DOCK6 expression correlates with proinvasion characteristics in a proteomic analysis of gastric cancer, suggesting that it may be a useful biomarker for tumor progression (102). It has also been demonstrated to promote resistance to chemotherapeutics and radiotherapy in gastric cancer (103). Similarly to DOCK1, DOCK6 inhibition could be antimetastatic, but targeting DOCK6 for therapeutic means may lead to off-target effects due to the high levels of DOCK6 expression throughout the body, as well as its clear role in neuronal functions.

### DOCK7 (KIAA1771, ZIR2)

DOCK7 exhibits dual specificity for RAC and CDC42 GTPases, with a slight catalytic preference for CDC42 (42). It is highly expressed in the brain, where it has roles in axon formation, polarity, and myelination (42, 48, 104, 105). It is also expressed widely throughout the body, with high levels noted in endocrine, eye, and reproductive tissues according to RNA expression data (Human Protein Atlas). A region of DOCK7 that includes the DHR1 was identified to bind the centrosome-associated protein TACC3, an interaction that was required for neural genesis and nuclear migration (106). More recently a role for DOCK7 in the migration of neuroblasts in developing mouse brains was also identified (107). DOCK7-controlled migration can also be pathological; it was identified to be required for human hepatocyte growth factor (HGF)-induced glioblastoma tumor cell invasion, with upregulated DOCK7 found in astrocytoma human glioblastoma compared with nonneoplastic brain (108). Interestingly, DOCK7 activity leads to migration in Schwann cells, and knocking down expression leads to increased myelination and differentiation (109). In keeping with observations that DOCK7 is crucial for neural processes, several studies have identified pathological links between premature stop codon mutations in DOCK7 that resulted in truncated protein and epileptic encephalopathy and cortical blindness (110, 111, 112). The majority of DOCK7 research has focused on its neural functions, despite its ubiquitous expression. Due to the recurring cytoskeletal remodeling and migratory capabilities, it can be hypothesized that DOCK7 will participate in similar processes in other cell types.

### DOCK8 (ZIR8)

DOCK8 is a CDC42-specific GEF (113). It is predominantly observed in the blood, bone marrow, and lymphoid tissues by RNA, while protein expression also indicates high levels in the lung and lower levels in other tissues (Human Protein Atlas). DOCK8 regulates a broad range of signaling cascades to control survival, motility, and synapse formation in lymphocytes; it is required for B-cell, T-cell, and natural killer cell function (114). In line with this, patients with large deletions in the DOCK8 gene experience increased susceptibility to pathogenic infections yet also exhibit allergic diseases, leading to great interest surrounding DOCK8 in the field of immunology, which has recently been reviewed in great detail (50, 115, 116). Recently, it has been shown that DOCK8 knockout in mononuclear phagocytes renders them highly susceptible to cell death when migrating through tissue, releasing inflammatory cytokines in the process (117). In the brain, DOCK8 deficiency leads to impaired activity of T-cells and microglia, with studies showing that impaired GEF activity results in ameliorated outcomes of experimental autoimmune encephalomyelitis (EAE), an animal model for multiple sclerosis (MS) (118, 119). Mutations in the DOCK8 gene have also been associated with autism disorders (116). Altogether, these findings demonstrate that, similarly to DOCK2, DOCK8 is a key element in the immune system. The potential for modulation of this protein in the treatment of MS is promising; however, potential immunodeficiencies will need to be considered.

### DOCK9 (KIAA1058, Zizimin-1, ZIZ1)

The DOCK-D subfamily, although not as well studied as other DOCKs, has been shown to have roles in the immune system and neurology (36, 48, 120). DOCK9 is a CDC42 GEF (35). It is widely and highly expressed throughout the body, including the brain; however, antibodies against two isoforms of DOCK9 indicated differing expression profiles (121). The role of DOCK9 in structural remodeling of cells was investigated in HeLa epithelial cells, where its expression reduced elongated cell morphology and led to increased filopodia and membrane ruffling (122). Although not reported as a substrate, overexpression of DOCK9 coincided with an increase in RAC1 activity. DOCK9 was recently implicated as a target for the alleviation of papillary thyroid carcinoma due to knockdown leading to reduced proliferation and migratory characteristics (123). Proteomic analysis indicated that DOCK9 interacts with neural AMPA receptors, which regulate synaptic plasticity (124). Such an interaction is worth investigation, as a reported point mutation in the AMPAR subunit GluA3 was shown to cause circadian rhythm disruption and intellectual disability (125). Knockout EAE mouse models of all DOCK-D family members were healthy and viable; DOCK9, however, had no reported change in phenotype, implying it is not involved in MS (120). Despite this result, the identification of structural remodeling as an important biological function of DOCK9 draws comparison to other family members, suggesting that further research is warranted in such areas as immune function. However, its ubiquitous expression increases the likelihood of off-target effects when inhibiting this protein.

### DOCK10 (KIAA10694, Zizimin-3, ZIZ3)

DOCK10 is a GEF for both CDC42 and RAC GTPases (42, 43, 44). Unlike DOCK9, it has tissue-selective expression noted in the brain, female reproductive tissues, skin, and lymphoid tissues (Human Protein Atlas), implying it may have a role in neurology and immunity. DOCK10 expression is reportedly induced by interleukin-4 (IL4), which activates signaling pathways involved in cell growth and differentiation (126). In chronic lymphocytic leukemia (CLL), IL4 prolongs the survival of CLL cells, which is linked to its known role in B-cell proliferation. This may mean that DOCK10 has a role in the downstream effects of IL4 in CLL cells and could therefore be of therapeutic interest. DOCK10 is also highly expressed in neurites of neuroblastoma cells (36). Recent studies suggest that its inhibition could lead to a milder phenotype of myelin oligodendrocyte glycoprotein (MOG)-induced EAE, suggesting that like DOCK8 it could be a target for treatment of MS (120). Interestingly, the EAE phenotype was not seen in DOCK9 or DOCK11 knockout mice, which could suggest that its unique binding partners or perhaps the ability of DOCK10 to bind RACs as well as CDC42 may be responsible for its role in neuroinflammation (120). Finally, recent reports implicate DOCK10 in aging processes, with knockout mice living longer than wild type (127). Overall, DOCK10 is a promising therapeutic target for several indications including leukemia, MS, and aging, warranting continued investigation.

### DOCK11 (Zizimin-2, ZIZ2)

DOCK11 is a CDC42-specific GEF that is highly expressed in B and T lymphocytes and has been shown to participate in B-cell homeostasis and differentiation mechanisms (94, 97, 128). It is also distributed among various tissues throughout the body (Human Protein Atlas). Similar to DOCK10, several studies have implicated DOCK11 in aging processes. Expression was observed to decrease in mouse lymphoid tissue, where it was reportedly involved in structural remodeling such as filopodial formation (129). It was also linked to Fcγ and TLR4 receptor engagement. Age-related decrease in DOCK11 levels in B-1 cells was related to reduced production of immunoglobulin M, an important factor in immune defense (130). This work was expanded upon by the observation that B-cell signaling is influenced by DOCK11, playing an important role in the generation of antigen specific B-cell populations in germinal centeres (131). In the EAE mouse model for assessing DOCK-D involvement in MS mentioned previously, macrophages from DOCK11 knockout mice exhibited limited migratory capability; however, the MS disease phenotype was not ameliorated by this loss (120). A commonality observed throughout the DOCK family, DOCK11 regulates cellular activities in the immune system, warranting broader investigation into its roles in the various homeostatic and pathological processes associated with inflammation.

### Family summary

As a family, the DOCK proteins share common features in their biological functions. Structural remodeling processes that result in motility, membrane organization, axon organization, myelination, and differentiation are all consequences of acting on the RHO GTPases. Therefore, DOCK proteins are often implicated in disease areas where the aforementioned processes can become pathological. Such examples include DOCK-controlled motility resulting in metastasis in cancer (DOCK1), deficient axon organization potentially resulting in a neurological disorder (DOCK4), or deficient immune cell migration resulting in immunodeficiency (DOCK8). In terms of therapeutic intervention, there is the promising possibility of inhibiting DOCK proteins to control metastasis and hyperactive immunity. One such promising example of the latter is identified in DOCK2, where inhibition is hypothesized to downregulate microglia activity and potentially ameliorate AD progression.

## Structural overview of the DHR2 domain

Prior to the recent cryo-EM structure of full-length DOCK2 in complex with ELMO1 and RAC1, structural investigation of DOCKs had been limited to recombinant domains with a focus on the catalytic DHR2 domain (Table 1). As detailed above, the DOCK proteins represent a promising class of proteins for pharmaceutical intervention in an array of diseases. There is therefore great interest in analyzing and identifying key mechanisms in the catalysis of GDP dissociation, as well as the structural and mechanistic differences between DOCKs and their GTPase substrates in order to achieve target selectivity.

**Figure 4:**
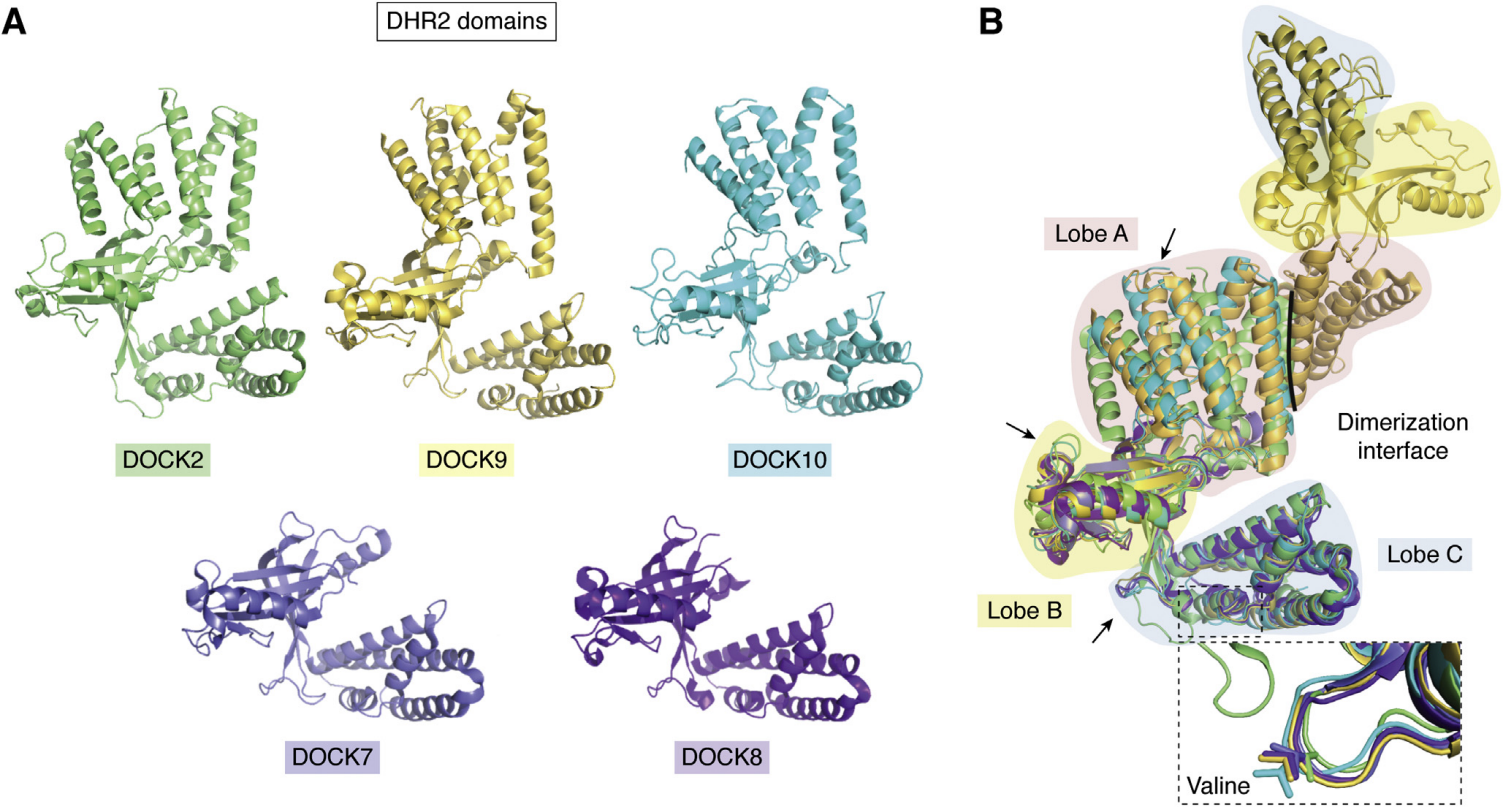
***A*, the individual DOCK DHR2 domains, represented as monomers.***B*, superposition of the DOCK DHR2 domains illustrates their similar overall fold that is segregated into three lobes A–C. Lobe A is made of 5 to 6 alpha helices depending on subfamily, and variations are observed in the interjoining loops between helices (examples highlighted by *arrows*). This lobe is the site of homodimerization (only DOCK9 dimer shown). Lobe A is not present in the structures of DOCK7 and 8 although predicted to be present based on sequence homology. Lobes B and C are responsible for GTPase substrate binding, discrimination, and catalytic activity. Variability is also observed in the interjoining loops between secondary structures (examples highlighted by *arrows*). Lobe C contains a universally conserved valine on the α10 insert (highlighted by *dotted box*), which is responsible for occluding the magnesium that is crucial for the GDP binding. DOCK2 PDB ID: 2YIN; DOCK7 PDB ID: 6AJ4; DOCK8 PDB ID: 3VHL; DOCK9 PDB ID: 2WM9; DOCK10 PDB ID: 6TM1.

Despite low sequence homology between DOCKs, DHR2 domains appear to adopt a similar fold that is characterized by three lobes A–C (Fig. 4) (35, 38, 41). From known DHR2 crystal structures, lobe A consists of five (DOCK9, 10) or six (DOCK2) antiparallel helices and is the site for homodimerization with a second DOCK protein (35, 41). Lobe A is not present in the recombinant DHR2 constructs used to generate the structural data for DOCK7 or DOCK8 (from *Mus musculus*) (42, 113). Therefore, there is a lack of structural insight into conservation of the helical arrangement, as well as the highly variable regions that exist between alpha helices 2 and 3. Lobes B and C are largely responsible for GTPase binding and GEF activity and are well resolved in X-ray crystal structures. Lobe B is formed by two orthogonal and antiparallel beta sheets in an arrangement that is largely conserved between DOCK family members, with variation observed in the interjoining helical regions (41). In DOCK7 it was identified as a sensor of differing switch 1 loop conformations in its dual substrate-binding mode (42). Lobe C, which consists of a 4-helical bundle, also exhibits high conservation in overall structure as well as in GTPase-binding residues. Lobes B and C together dictate GTPase substrate binding, with specificity and discrimination in part determined by DHR2 complementarity with a hydrophobic residue such as the Phe or Trp at position 56 and Ala or Lys at position 27 in RHO GTPases (41). Equivalent residues DOCK2-Tyr1368 and DOCK7-Ile1836 were hypothesized to confer specificity for RAC1 and CDC42 respectively. However, a mutant DOCK7 Ile1836Tyr did not replicate DOCK2 specificity for RAC1, and other factors such as flexibility in lobe B were also hypothesized to contribute to substrate selection (42). Lobe C also contains a helical insert (α10) that houses a critical and universally conserved valine residue that serves as a sensor for nucleotides when bound to GTPases and is key to the mechanism by which GEFs induce GDP dissociation.

## The DHR2 mechanism of action

**Figure 5:**
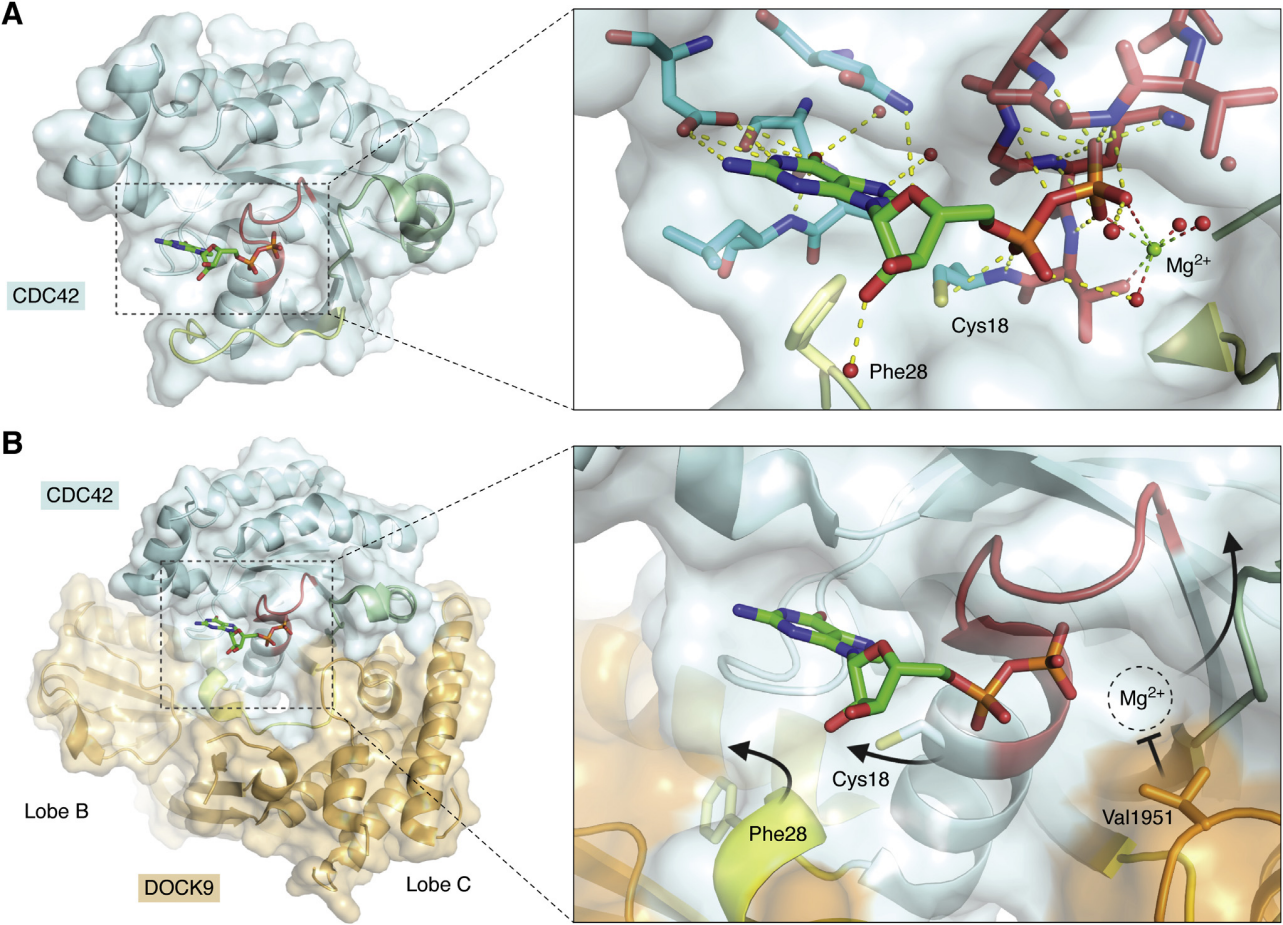
***A*, GDP (*green sticks*) binds to CDC42 (*cyan surface*, *sticks*) through an extensive network of intermolecular interactions.** Hydrogen bonding and salt bridges are indicated by *dashed yellow lines*. Most interactions are concentrated around the nucleoside-binding region (*cyan sticks*) and the phosphate-binding loop (P-loop; *red sticks*). *Right*, crucial CDC42 interactions in the GEF -induced GDP dissociation mechanism are highlighted: 1. Phe28 is located in the switch 1 (*yellow cartoon*, *sticks*); 2. Cys18 is proximal to the P-loop; 3. Mg^2+^ (*small green sphere*) is chelated by GDP phosphates, the P-loop, and several water molecules (*small red spheres*). (CDC42 PDB ID: 1A4R). *B*, *Left*, DOCK9 (*Orange surface* and *cartoon*) binds to GDP-bound CDC42 (*blue surface*) *via* lobes B and C. For orientation, this representation is the underside of the view presented in Figure 4. *Right*, close-up of the CDC42 GTP-binding site when bound to DOCK9. Only the critical features involved in the GDP dissociation mechanism are presented. Phe28 and Cys18 rotate away from the GDP molecule, while the DOCK9-Val1951 juts into the active site and occludes Mg^2+^ binding. The *dotted sphere* indicates where Mg^2+^ binding was before DOCK9 binding. The cumulative result of these conformational changes leads to significantly decreased affinity between CDC42 and GDP. (DOCK9:CDC42 PDB ID: 2WMN). Notably, the switch 2 region (*green ribbon*) of CDC42 is unchanged on DOCK binding.

The earliest structures of a DOCK protein detailed a three-step mechanism by which the DOCK9-DHR2 domain elicits GDP dissociation from CDC42 (Fig. 5) (35). In its inactive state, CDC42 binds to GDP with extremely high affinity that is mediated through an extensive network of intermolecular interactions (Fig. 5*A*) (132). Several residues in the hydrophilic binding pocket are responsible for hydrogen bonding to the base moiety of GDP, while a noteworthy Phe28 from the switch 1 loop contributes a hydrophobic interaction with the guanine nucleobase. The phosphate-binding loop (P-loop) binds to the GDP phosphate moiety, which together chelate an Mg^2+^ ion, further contributing to the high-affinity interaction. Upon association between CDC42 and the DOCK9-DHR2 domain, three structural features cause a reduction in affinity between CDC42 and GDP (Fig. 5*B*). First, the CDC42 switch 1 loop is displaced, which results in abrogation of the interaction between Phe28 and the GDP base. Secondly, Cys18 in the P-loop is rotated away from the GDP β-phosphate, disrupting a hydrogen bond between the SH and phosphate. Finally, the aforementioned α10 helical insert present in lobe C of the DOCK9-DHR2 extends into the CDC42-GDP-binding site, placing the nucleotide sensing DOCK9-Val1951 such that it directly occludes Mg^2+^ binding. This is predicted to cause a dramatic affinity reduction due to interruption of the highly coordinated interaction that includes the P-loop and GDP, as well as loss of the Mg^2+^ mediated neutralization of the negatively charged nucleotide phosphates. This prediction was strengthened by the observation that replacement of the Val1951 with an alanine severely reduces DOCK GEF activity. The importance of this valine is further supported by its conservation throughout the DOCK family (Fig. 4). Together these structural features encourage the release of GDP and allow a GTP molecule to bind, which results in an active GTPase that can participate in cellular signaling.

## Targeting GTPases *via* their complex with DOCKs

**Figure 6:**
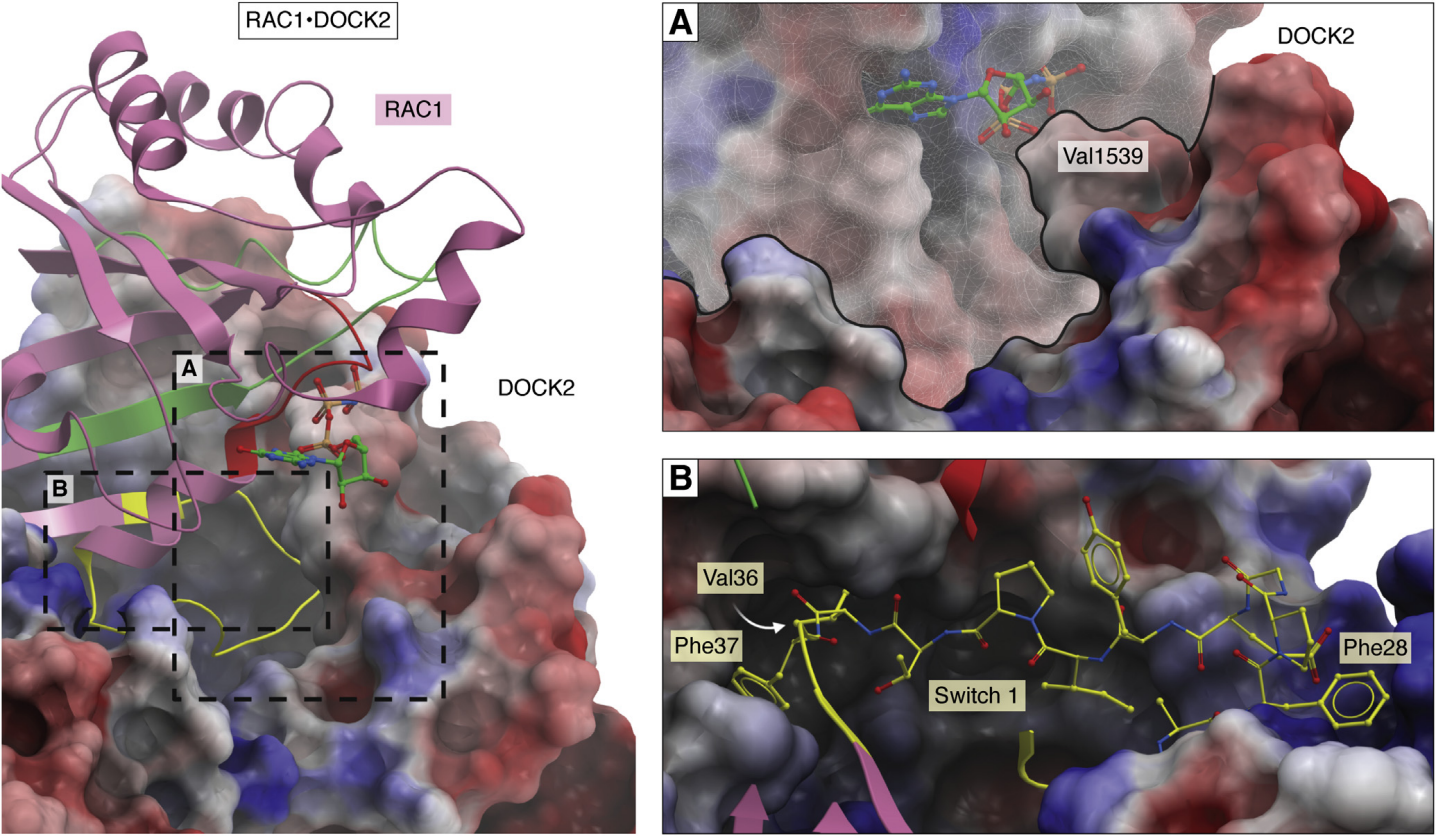
**Potential options for drugging the DOCK-GTPase complex.***Left*, overview of the DOCK2-RAC1 complex (electrostatic surface, *pink ribbon* respectively; PDB ID: 2YIN). *A*, an electrostatic representation of the DOCK2-RAC1 interface, overlaid with RAC1-GDPNP (PDB ID: 3TH5) to visualize the GTP (*green sticks*) binding site. The RAC1 surface is highlighted by the *transparent white mask*. The nucleotide sensing Val1539 that is unique to DOCK proteins binds adjacent to the β and ɣ-phosphates of GTP. Compounds that bind the P-loop may benefit from an adjacent hydrophobic group for complementarity to this residue. This could also confer selective for DOCK2 over DH GEFs. The interface visible here is predominantly due to the switch 1 loop interacting with lobe C of the DOCK2-DHR2 domain. Due to variation observed in this region, compounds that bind to this region could potentially achieve selective targeting between DOCK subfamilies. *B*, the switch 1 loop (*yellow sticks*) of RAC1 (*pink ribbon*) binds into a hydrophobic groove in lobe C of the DOCK2-DHR2 domain (electrostatic surface). Complementarity to this groove is observed with Val36 and Phe37, highlighting a potential target for inhibition at the protein–protein interface of the complex. The Phe28 involved in binding the nucleotide also binds into a pocket on lobe C of the DOCK2-DHR2. For orientation, the view is looking through RAC1 with the switch 2 and the P-loop (*green*, *red ribbon* respectively) visible at the *top*. Electrostatic surfaces generated by ICM Molsoft.

Aside from their crucial role in cellular regulation, the activity of GEFs also represents an excellent opportunity for therapeutic intervention. The extremely high affinity with which guanosine nucleotides bind to GTPases ordinarily means that small-molecule inhibitors are unlikely to be able to compete and displace the substrates effectively (133). However, GEF activity induces a conformation wherein GDP-binding affinity is greatly reduced, coinciding with a large increase in dissociation rate, making substrate displacement much more feasible (134). X-ray crystallography is an amenable technique for this purpose, as it is possible to trap GTPases in this state upon crystallization of the GTPase-GEF complexes, allowing for compound screening *via* a platform such as XChem at Diamond Light Source as well as facilitating subsequent chemical design (135, 136). This rationale was used in the development of a Target Enabling Package for the DH GEF Kalirin and RAC1 recently developed by the Structural Genomics Consortium (137). It is important that such experiments are accompanied by in-solution experiments to confirm the crystallographically determined mode of binding. Through this approach it would be feasible to design inhibitors that bind specifically to the unique site, which arises upon GEF-GTPase complexation (Fig. 6*A*). Indeed, a reported inhibitor for DOCK5 was observed to cause a nonfunctional and inhibitory complex; however, the atomic binding mode for this compound is not yet publicly known (91). Additional possibilities for inhibition of GTPase pathways include inhibiting the broad GEF-GTPase protein–protein interaction at lobes B or C of the DOCK2-DHR2. For example, in the DOCK2-RAC1 complex, switch 1 loop residues Val36 and Phe37 of RAC1 bind into hydrophobic grooves in lobe C of the DOCK2 DHR2 domain (Fig. 6*B*). Such regions are potential targets for disruption of the protein–protein interaction. Indeed, small structural differences at this region such as the aforementioned complementarity between residues 27 and 56 on GTPases and the DHR2 domain are enough to control the stringent discrimination for DOCK substrate selection. Accordingly, chemical interrogation of this region could potentially disrupt complex formation, preventing the ability for GTPases to return to an active state once bound to GDP. A similar protein–protein interaction inhibitor approach has been applied to the RAS GEF SOS1 as potential cancer therapeutics (11, 12). Finally, targeting the DHR1 phospholipid-binding site could prevent complex localization to the membrane.

The various crystal structures of DOCK-GTPase complexes (Table 1) reveal that the conformation of the GTPase nucleotide-binding site alters in a manner that varies between GEF partners, implying the possibility of selective compound design. The DOCK mechanism of eliciting GDP dissociation is distinct from the canonical DH-domain containing GEFs, where Mg^2+^ occlusion is typically accomplished by eliciting displacement of the GTPase switch 2 loop (35). As previously mentioned, the universal nucleotide sensing Val (Fig. 4, Residue 1951 in DOCK9, Fig. 5*B*; 1539 in DOCK2, Fig. 6*A*) is unique to the DOCK subfamily. This valine protrudes into the GTPase nucleotide-binding site proximal to the β and ɣ-phosphates of GTP, which bind to the P-loop backbone through electrostatic and hydrogen-bonding interactions. Inhibitor design aiming to exploit these potentially high-affinity contacts may be accompanied by an adjacent hydrophobic group to bind the valine and contribute further to binding affinity. Such an inhibitor binding to this residue or those on the nearby α10 insert could potentially prevent the transient DOCK GEF-GTPase complex from dissociating, preventing the propagation of signaling. Selectivity for DOCK-GTPases over GTPases alone or with other GEF complexes could prevent potential off-target effects of targeting these pleiotropic proteins.

**Figure 7:**
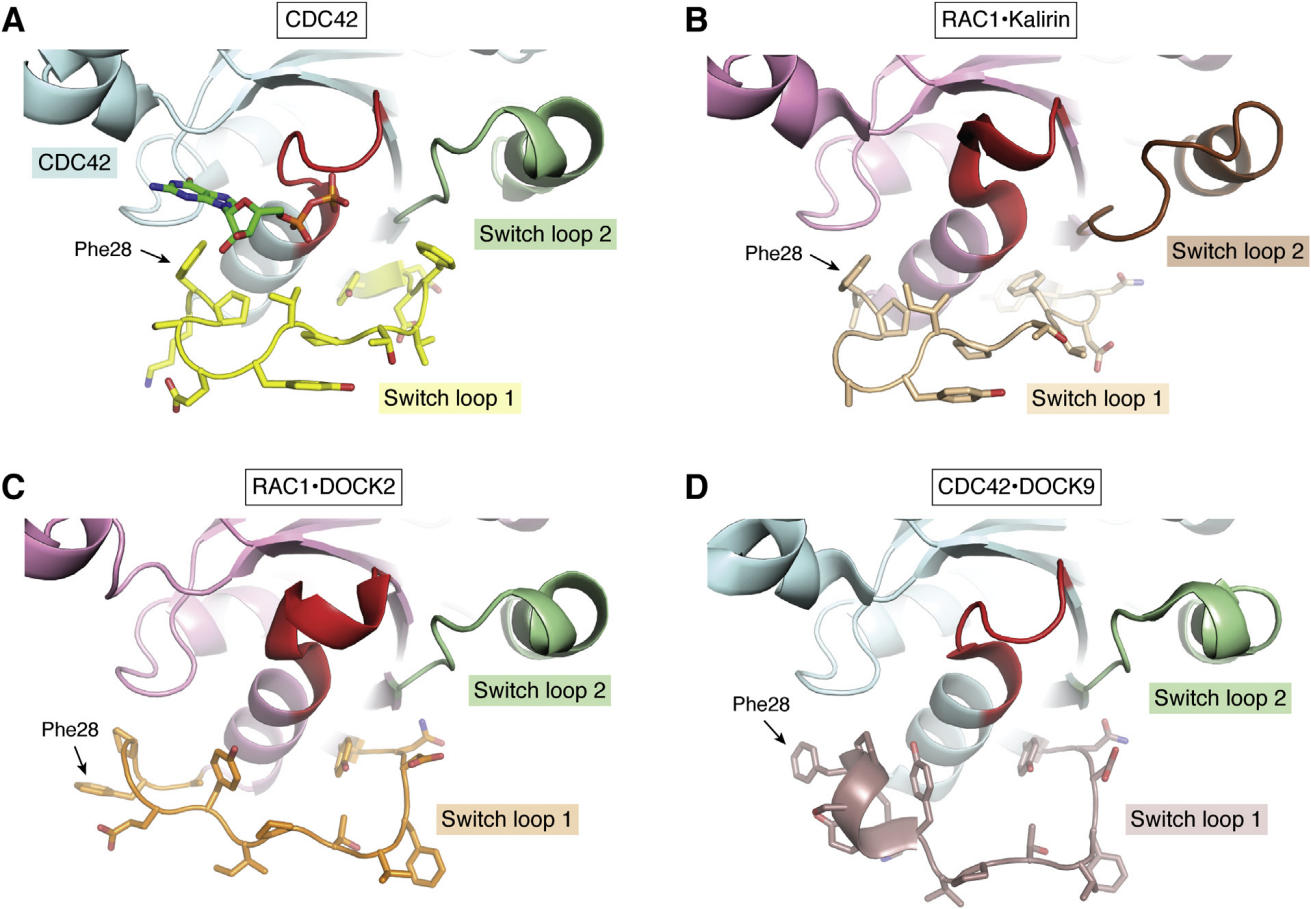
**Illustration of variation in the switch loops of RAC1 (*pink cartoon*) and CDC42 (*cyan cartoon*).** GDP (*green sticks*) and the P-loop (*red cartoon*) are visible for orientation. Switch loops are colored dependent on their conformation as a result of complexation with a GEF. GEFs are not depicted for viewing clarity in panels *B*–*D*. *A*, the switch loop conformations in CDC42 when bound to GDP but not complexed with a GEF. These conformations vary minimally between RHO GTPases when bound to GDP, CDC42 is used to represent all here. PDB ID: 1A4R. *B*, Kalirin elicits altered switch 1 (*beige cartoon* and *sticks*) and switch 2 (*brown cartoon*) loop conformations compared with those in the noncomplexed GTPase represented in panel *A*. Of note, however, is the similarity observed in residue Phe28. Kalirin is representative of all DH GEFs, which elicit similar switch loop conformational changes throughout the family. PDB ID: 5QU9. *C*, DOCK2 also elicits an altered switch 1 loop (*orange cartoon*) compared with GTPase alone (*yellow cartoon*, panel *A*). The switch 1 loop is also distinct from the conformation observed when RAC1 is bound to Kalirin (panel *B*), exemplified by the highlighted Phe28 residue. The switch 2 loop conformation is identical to that of CDC42 alone and is thus distinct from that observed in the RAC1-Kalirin structure. PDB ID: 2YIN. *D*, finally, the CDC42 switch 1 loop conformation is different again in the DOCK9 complexed structure. Here, DOCK9 is representative of all known DOCK-C and D GEFs, which elicit the same conformational change. These differences highlight the potential for selective targeting of GTPases when bound to DH GEFs or different DOCK families. PDB ID: 2WMN.

In contrast to the DH GEFs such as Kalirin, the DOCK GEFs do not induce changes in the switch 2 region of their GTPase substrates and conformational changes are confined to switch 1 (Figs. 5 and 7, *A*–*D*). Targeting the GTPase switch 1 loop is a promising avenue for inhibitor design. One could take advantage of the guanine nucleobase-binding pocket to provide an anchor for inhibitors to bind the DOCK-GTPase complex. Here, the switch 1 loop undergoes conformational changes to form an interface that is a topologically wide groove, characterized by complementarity between the proteins through charged and hydrophobic regions (Fig. 6*A*). Switch 1 conformational changes occur in a manner dependent on the interacting DOCK. The sequence and structure of the DHR2 B-C lobes of DOCK2 and DOCK9 result in nonidentical switch 1 loop conformations in RAC1 and CDC42 respectively (Fig. 7, *C* and *D*) (41). This phenomenon appears to be consistent within related DOCK subfamilies, with DOCKs 7, 8, 9, and 10 (DOCK-C, D members) eliciting similar switch 1 conformations in their partner GTPases whether it is CDC42 or RAC1, whereas DOCK2 (DOCK-A subfamily) elicits a different conformation in the switch 1 loop of RAC1. This is again in contrast to DH GEFs, which elicit similar conformational changes in their GTPases substrates throughout the family. The DOCK-induced conformational changes result in slightly altered GTPase pockets, including differing orientations of the aforementioned Phe28 in CDC42 and RAC1, an important residue in binding the GTPase nucleotide substrate (Figure 5, Figure 6, Figure 7). A small molecule designed in a way that specifically binds to this induced conformation may be selective for the DOCK-GTPase complex over the GTPase alone, the DH GEF-GTPase complex, or even between different DOCK subfamilies, leading to an even higher degree of selectivity.

## Conclusions

Acting on the universal and pleiotropic GTPases, the DOCK GEFs are responsible for controlling many essential biological processes, particularly structural remodeling of cells leading to migration, differentiation, and proliferation in many cell types. This family of proteins is consequently implicated in various diseases, ranging from cancer to neurodegeneration. Distinct structurally and mechanistically from the larger DBL homology (DH) family of RHO GEFs, DOCKs present an opportunity for selective modulation of GTPase pathways with small molecules. The transitioning GTPase exhibits a GEF-induced reduction in affinity for its substrate nucleotide. Thus, the GEF-GTPase complex may present a feasible opportunity to compete with small-molecule inhibitors instead of the GTPase alone. The pockets forming in the DOCK-GTPase complex appear to have structural differences, which bodes well for the development of inhibitors selective for certain DOCK-controlled signaling cascades, which differ between the families. While there is a growing body of literature concerning the biological function and structural mechanism of DOCK proteins, there remain gaps in current knowledge of this field. The vast majority of investigation into RHO GTPases has been limited to the RAC1, CDC42, and RHOA proteins, a small subset of the RHO family. To further our understanding of the RHO GTPase pathways, it is imperative to apply similar methodologies to those presented in this review to the remaining members of the RHO GTPase family, including the RhoA, RhoBTB, RhoD/F, RHOU/V, and RND subfamilies and their cognate DOCK GEFs. Finally, there is currently a limited number of small-molecule inhibitors with which to probe the activity of the DOCKs, such molecules would provide integral insight into their modulation for treating disease and provide starting points from which to design and discover new drugs.

## Conflict of interest

The authors declare that they have no conflicts of interest with the contents of this article.
